# Impact of proprioceptive cervical dizziness in chronic neck pain syndromes on gait and stance during active head-turn challenges

**DOI:** 10.1007/s00415-024-12711-8

**Published:** 2024-10-15

**Authors:** D. Huppert, T. I. Tsai, S. Richter, K. Dunker, J. Gerb, B. Wegener, R. M. Zwergal, M. Wuehr, T. Brandt

**Affiliations:** 1 German Center for Vertigo and Balance Disorders, University Hospital, LMU University Hospital, Ludwig-Maximilians-Universität München, Marchioninistrasse 15, 81377 Munich, Germany; 2 Department of Neurology, University Hospital, LMU University Hospital, Ludwig-Maximilians-Universität München, Munich, Germany; 3 Musculoskeletal University Center Munich, University Hospital, LMU University Hospital, Ludwig-Maximilians-Universität München, Munich, Germany; 4 Interdisciplinary Pain Center, University Hospital, LMU University Hospital, Ludwig-Maximilians-Universität München, Munich, Germany

**Keywords:** Cervical dizziness, Chronic neck pain syndrome, Gait, Balance, Cervical rotation

## Abstract

An earlier observational study described selected patients with acute neck pain syndrome, who experienced short bursts of cervical vertigo elicited by rapid head movements. The current study on a larger cohort of 20 patients with chronic or frequently recurring neck pain syndrome and age-matched controls focused on two major questions: (1) Can head movements in subjects with exacerbations of neck pain and restrictions of neck mobility also elicit bursts of vertigo? (2) What is the impact on postural balance measured by analysis of body sway and locomotion? A detailed questionnaire was applied, posture and gait were evaluated by use of instrumented posturography—and gait analysis with and without slow or rapid horizontal head rotations in the yaw plane with and without sight/visual input. All patients reported some or frequent episodes of dizziness in the range of seconds only elicited by rapid, not by slow head movements. Postural sway in patients was unremarkable in undisturbed conditions without head movements, but specifically increased by rapid but not slow head turns. The latter is best explained by the lack of continuous control of velocity and amplitude of saccadic head movements. Gait analysis revealed a slowed and cautious gait pattern already at undisturbed condition that was even exaggerated during rapid head turns. These observations demonstrate that chronic or recurrent neck pain is associated with episodic experiences of dizziness and above results in both chronic and episodic alterations of stance and gait that resemble those described for patients with phobic postural vertigo/persistent postural perceptual dizziness, a functional gait disorder.

**Supplementary Information:**

The online version contains supplementary material available at 10.1007/s00415-024-12711-8.

## Introduction

Cervical dizziness (CD)—reality or fiction—is the subject of a long-standing interdisciplinary debate. Supporters believe it to be one of the most common causes of dizziness with vertigo, disorientation, and disequilibrium confirmed by a range of signs and symptoms. Their opponents reject the diagnosis because of the lack of a reliable clinical test to distinguish it from other pathological forms of episodic dizziness [10, 23, 48]. The Bárány Society Classification OverSight Committee takes the view “that the evidence supporting a mechanistic link between an illusory sensation of self-motion (i.e. vertigo—spinning or otherwise) and neck pathology and/or symptoms of neck pain—either by affecting the cervical vertebrae, soft tissue structures or cervical nerve roots—is lacking” [37]. On the other hand, it is commonly acknowledged that cooperative interactions between proprioceptive neck afferents and head-mounted vestibular and visual systems cues control the coordination of eye, head, and body movements in space. They transform registered rotations and translations they sense relative to the direction of motion (i.e., postural orientation) and fine-tune the body’s center of gravity (i.e., static and dynamic stability) [12]. This implies that stimulation or functional distortions of these structures could produce CD. In fact, unilateral local anesthesia of the upper dorsal cervical roots has been shown to induce ataxia and nystagmus in animals and ataxia without nystagmus in humans (Table 1; for review see [1, 17]). This table listing a number of various experimental animal and human studies on different species we considered as relevant since the controversial articles on cervical vertigo often use one or several results for their discussions of hypothetical pathophysiology.

**Table 1:** Ataxia, disequilibrium, and nystagmus in experimental cervical vertigo

Species	Experimental methods	Signs and symptoms	Authors
Dog, cat, rabbit, horse	Surgical damage to neck muscles	Ataxia similar to hemicerebellectomy	Longet (1845) [31]
Rabbits	Damage to neck structures	Ataxia	Bernard (1865) [6]
Humans	Local anesthesia of deep postero-lateral neck tissue	Increased ipsilateral and decreased contralateral extensor muscle tone with gait deviation and past-pointing toward injected side; no nystagmus	Barré (1926) [3] Hinoki and Kurosawa (1964) [25] DeJong et al. (1977) [17] Dieterich et al. (1993) [18]
Rabbits	Upper cervical root section	Positional nystagmus	Biemond (1939, 1940) [7, 8]
Rabbits, cats, Rhesus monkeys	Local anesthesia of neck afference	Positional nystagmus, species-specific, most pronounced in rabbits, less in cats, subtle in Rhesus monkeys	Cohen (1961) [16]
Humans	Vibration to neck muscle tendons	Perceptual and postural illusions	Goodwin et al. (1972) [22] Tayler and McCloskey (1991) [42] Karnath (1994) [28]

Clinically one would expect CD—if it exists in non-specific neck pain syndromes—would preferably manifest with head rotations. In a first observational study, a small number of the patients studied with acute neck pain and mainly unilateral constraints of head rotation spontaneously complained about spells of dizziness or vertigo elicited by head movements [13]. The descriptions of their complaints were very similar: (1) apparent surround motions or short body perturbations for a fraction of a second with postural unsteadiness; (2) attacks evoked only by rapid, not by slow head rotations, when standing or during locomotion; (3) attacks subsided spontaneously along with the recovery from the neck pain. This syndrome was referred to as head motion-induced spells of cervical vertigo in patients with acute neck pain [13].

**Fig. 1:**
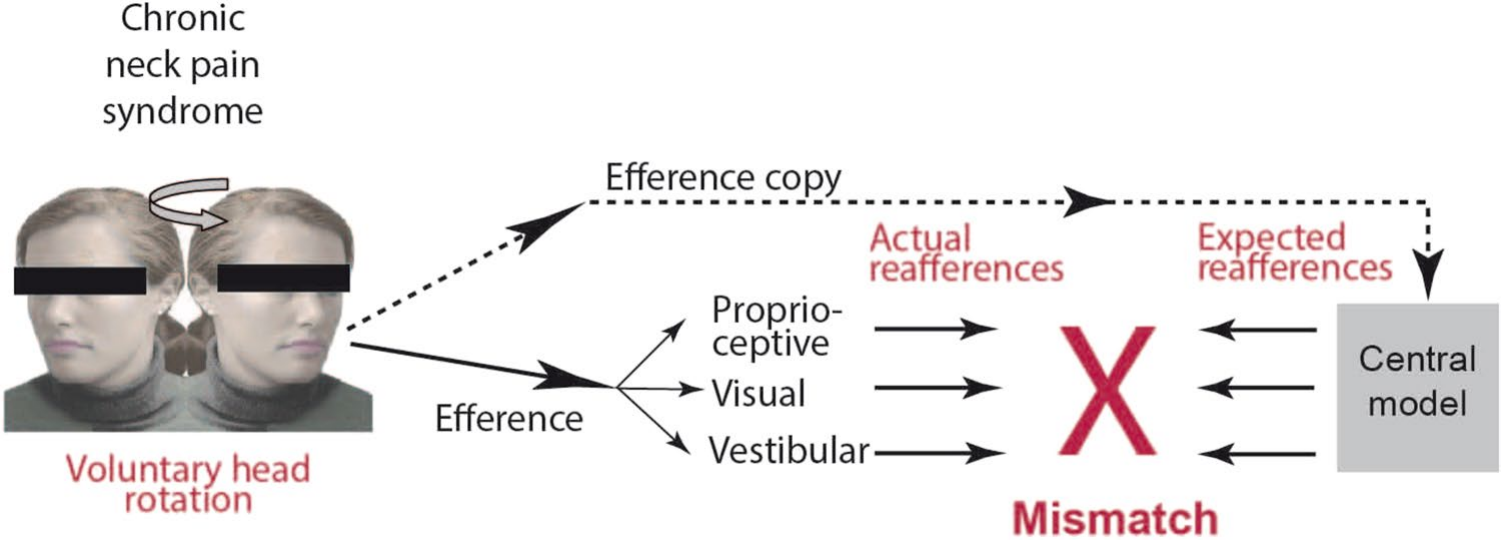
Diagram showing the neural mismatch concept in chronic neck pain syndrome. Voluntary head movements (efference) stimulate the proprioceptive neck muscle and tendon receptors, the visual and vestibular system (actual reafferences). The effferency releases a multisensory pattern from an internal central model programmed by earlier sensorimotor experiences. If the intended head movement falls shorter by muscle stiffness and pain a mismatch occurs which can cause spells of dizziness and postural instability (mod. after [13])

Based on the sensorimotor mechanism for the perception of space constancy during active movements (i.e., ‘the efference copy and reafference principle’ after von Holst & Mittelstaedt [44]) these spells of vertigo were proposed to result from a head-motion induced misalignment of sensorimotor integration between real versus intended head-on-trunk movements (elaborated in [10, 13]) (Fig. 1). Briefly, the logic is that if neural mismatch results in an inaccurate kinesthetic feedback of head movements, head–neck awareness during active locomotion and, subsequently, the maintenance of postural balance may also be affected, leading to unsteadiness, light-headedness and/or disorientation [27, 30, 36]; for review see [40]. This led the way for further theoretical and experimental publications of the so-called ‘internal model theory’ [9, 45].

The finding of short vertigo attacks elicited by rapid head movements in patients with acute neck pain prompted us to look for similar symptoms in patients with chronic or chronically recurring neck pain with and without significant constraints of head movements. A detailed patient history on the association of neck pain and disequilibrium combined with a standardized questionnaire was used. In addition, the possible impact of the painful neck stiffness on stance and gait control with and without slow and rapid head rotations in the yaw plan was analyzed by use of instrumented posturography and gait analysis. If chronic painful cervical syndromes cause pathological alterations of stance and gait during head movements, a further question would be how specific these alterations are with respect to the differential diagnosis from other gait disorders.

## Methods

### Participants

Twenty patients with varying chronic neck pain with a minimum duration of 6 months (14 females, mean age 49.7 ± 14.7 years) took part in the study and underwent a comprehensive clinical assessment (described below). They were referred to the study as outpatients from either the German Center for Vertigo and Balance Disorders or the Interdisciplinary Pain Center of the LMU Hospital Munich, Germany. Inclusion criteria were adult patients with varying levels of head mobility restriction due to cervical spine syndrome (because of muscle tension, osteoarthritis, blockage of a facet joint) and their ability to co-operate. Exclusion criteria were other neurological, orthopedic, or vestibular disorders assessed by clinical, neuro-otological, and orthoptic examinations including caloric irrigation test, video head impulse test, and subjective visual vertical test (SVV). Thirteen age-matched healthy subjects (HS; 6 females; aged 47.7 ± 14.4 years) served as a control group for the 13 patients with CD who underwent specialized instrumental tests (stance and gait analysis with head motion challenges; see below).

All participants provided their written consent to the analysis of the collected data. The experimental procedure was approved by the ethics committee of the Ludwig-Maximilians-Universität Munich’s Medical Faculty and complied with the ethical standards of the Declaration of Helsinki.

### Clinical assessment

On the day of examination, each patient underwent a structured clinical interview, which included a detailed history taking with specific questions about the dizziness complaints, i.e. type, trigger, duration, frequency, time course, individual and socioeconomic impact. Patients were also required to self-score their CD symptoms using a standardized Neck Pain and Disability Scale (selected and modified questions, see Table in Supplemental Material). Unless otherwise stated, all responses on the Neck Pain and Disability Scale were rated on a scale ranging from 0 (weakest manifestation) to 5 (strongest manifestation). Furthermore, the patients underwent a clinical orthopedic examination with determination of each patient’s head mobility in both yaw- and pitch-planes (in degrees), and a neurological examination with special focus on ocular motor and orthoptic tests. In addition, postural function of patients was determined using clinical routine posturography in conjunction with a dedicated neural network analysis (see details in [29]). Briefly, in this assessment the sway pattern of the patients is measured under 10 increasingly challenging stance conditions (including standing with eyes closed, on foam, with head reclination, and in tandem stance in different combinations) and quantified based on established sway parameters. Subsequently, using an artificial neural network, an automated diagnostic assignment to healthy stance behavior or four common postural disorders (including functional stance disorder) is made [2].

### Assessment of stance and gait function

In a subgroup of 13 CD patients (11 females; mean age 47.4 ± 9.7 years, range: 28–59 years old) and 13 age-matched healthy subjects, we also examined the impact of CD on stance and gait performance by posturography [29] and automated gait analysis [2].

The impact of CD on postural stability was assessed on a **stabilometer platform** (Type 9261A; Kistler; Winterthur, Switzerland) during six stance conditions: (1) free standing with eyes open and looking straight ahead (EO); (2) standing with eyes closed (EC); (3) standing with EO while performing slow continuous head turns; (4) standing with EC while performing slow continuous head turns; (5) standing with EO while performing fast head turns; (6) standing with EC while performing fast head turns. Conditions with slow head turns required participants to make slow, pendulum-like horizontal head rotations from shoulder-to-shoulder with self-preferred velocity (i.e., continuous left-to-right head movement) in the yaw-plane. Conditions with fast head turns required participants to make rapid midline-to-shoulder head rotations (from straight ahead to horizontal targets on the wall 50° to the right or left in yaw; alternating to the left and right side), returning their gaze back to the midline after each movement. These fast eye-head-turns were saccadic, i.e., no velocity and amplitude control during the voluntary movement. The timing of the slow and fast head turns was dictated by the audible clicks of a metronome set at 20 beats per min. For the slow head turn paradigm, participants were instructed to steadily turn their head from side to side during the interval between the beats; for the fast head turn paradigm, participants were asked to perform a quick head turn at each beat while remaining stationary in the inter-beat interval.

Each stance condition was recorded for 30 s at 40 Hz. The amount of body sway was quantified based on the radial center-of-pressure (CoP) trajectory in the time domain by (1) sway root-mean-square (RMS, mm) and (2) sway velocity (mm/s) in various frequency domains by spectral energy magnitudes: (3) the low-frequency band (0.1–2.4 Hz), (4) the medium frequency band (2.43–3.5 Hz), and (5) the high-frequency band (3.53–8 Hz). Spectral energy magnitudes were computed after application of a Hamming window using discrete Fourier analysis.

The impact of CD on gait was assessed using a 6.7 m-long **pressure-sensitive carpet** (GAITRite®, CIR System, Sparta, NJ, USA) at 120 Hz during two conditions: (1) walking at individually preferred speed and (2) walking at preferred speed while performing fast head turns. In analogy to the posturographic assessment, the condition with fast head turns required participants to make rapid midline-to-shoulder head rotations (alternating to the left and right side) at every third step. Each walking condition was repeated four times to collect enough strides for further analysis. Gait performance was characterized based on five established gait metrics that represent five previously established domains to comprehensively characterize a gait pattern [19]: gait velocity, swing phase, stride time variability (computed by the coefficient of variation, CV), stride time asymmetry, and base of support.

In addition, functional mobility and gait performance were evaluated by two established clinical tests: the Timed up and Go (TUG) test (Podsiadlo et al. [33]; Tinetti [43])—a test that measures how quickly one can stand up, walk three meters, turn around, walk back, and sit down—and the Functional Gait Assessment (FGA) [46].

### Statistical analysis

Descriptive statistics are presented as means ± SD. Differences in gait and stance performance between HS and patients with CD were evaluated by a repeated-measures multivariate analysis of variance (MANOVA) with assessment condition and group (patients vs. controls) as factors. Post hoc Bonferroni adjustments were used to correct for multiple comparison in each analysis. Potential associations between abnormal findings in the gait and stance performance of patients and the outcomes of the clinical questionnaire were examined using Spearman’s rank correlation coefficient. Results were considered significant at* p* < 0.05. Statistical analysis was performed using SPSS (Version 26.0, IBM Corp., USA) and JASP (Version 0.18.1, https://jasp-stats.org/).

## Results

## Analysis of the clinical questionnaire and clinical neurological examination

Of the 23 patients recruited for the study, three had to be excluded from the final analysis because of other neuro-otological disorders (vestibular migraine, cerebellar disease) or the inability to organize a follow-up-visit. Of the remaining 20 patients (14 females, 49.6 ± 12.4 years; males 49.8 ± 19 years), 13 described bilateral and 7 unilateral neck pain symptoms with an average overall duration of the pain episodes being of over 5 years (63.5 ± 73.9 months). Nineteen of the 20 patients completed the questionnaire.

Analysis of the neck pain and disability scale.

**Fig. 2:**
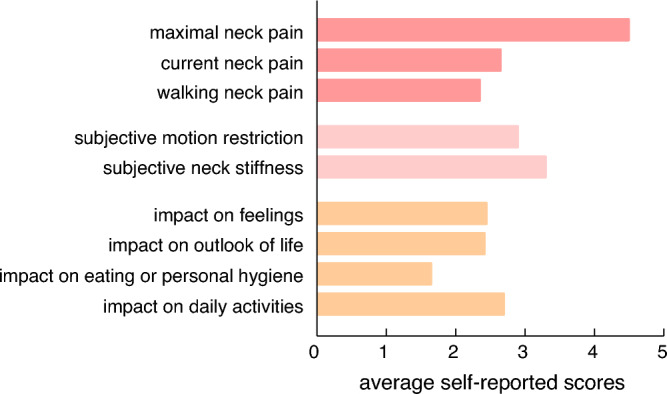
Average self-reported reported scores: pain scores (red colors), subjective cervical spine impairment scores (pink colors), and impact scores of quality of life (orange colors)

Unless otherwise stated, all responses on the Neck Pain and Disability Scale were rated on a scale from 0 (weakest manifestation) to 5 (strongest manifestation; see Suppl. Table). All patients felt impaired to varying degrees by their cervical syndrome (Table 2 and Fig. 2). On the day of the examination, the patients reported an average score of 2.65 ± 0.99 for neck pain. The average maximum score during the course of the disease was 4.50 ± 0.76 and the average pain score during locomotion/walking was 2.35 ± 1.46. Even though the impact on daily life due to neck pain was rated as 2.70 ± 2.00, personal care routines, i.e., eating, personal hygiene such as washing, dressing etc., were minimally affected (1.65 ± 1.79). The mean impact of neck pain on the patients’ outlook on life and feelings were 2.42 ± 2.14 and 2.45 ± 1.85, respectively. Finally, subjective neck stiffness was 3.30 ± 1.30, and subjective movement restriction 2.90 ± 1.41.

**Table 2:** Patient data gathered from the (Suppl. Table) questionnaire

Patient demographics	
*N*	20 (14 females)
Mean age (in years)	49.7 ± 14.7
Cervical spine complaints	
Bilateral	13
Unilateral	7
Mean duration of cervical spine complaints (in months)	63.5 ± 73.9
Dizziness^a^	
Short bursts of dizziness?	19 (13 females)
During head rotation?	10 (bidirectional: 5, unidirectional: 5)
During head flexion?	7 (inclination:5, reclination: 1, both: 1)
While sitting?	4
While lying down?	4
Reported dizziness characteristics^b^	
Drowsiness	12
Rotatory vertigo	8
Unsteadiness	8
Perturbations	1
Duration	
Seconds	9
Minutes	4
Hours or permanent	6
Frequency	
Daily	4
More than 5 times	11
2–5 times	3
Once	1
Individual impact	
Fear of falling	8
Significant discomfort	4
Fear of chronification	2
Avoidance behavior	10
Subjective impairment due to dizziness	12
Mean impairment score (1–10)	3.33 ± 0.94
Socioeconomic impact	
Sick leave	4
Prior doctor consultation	14
Consulted specialists	
Neurology	11
Orthopedic	11
GP	9
ENT	7
Ophthalmology	4

^a^Only 19 patients filled out this part of the questionnaire

^b^Characteristics not mutually exclusive

### Analysis of specific questions on cervical syndrome and dizziness

All nineteen patients who complained of dizziness associated with their neck pain syndrome had experienced sudden, brief episodes of dizziness or unsteadiness in the event of neck pain and restricted head movements, particularly with rapid head movements. Fourteen of these nineteen patients only experienced dizziness in the acute stage of sensing neck pain while the remaining five patients also reported dizziness to linger even after the acute neck pain subsided. In ten patients, attacks occurred predominantly during horizontal head rotations (evenly distributed for bilateral and unilateral movements); in seven, they were elicited by vertical head movements in the pitch plane (5 head flexion, 1 head declination, 1 in both directions); four reported this while sitting, and four while lying or lying down. The quality of dizziness was described as drowsiness (*n* = 12), spinning vertigo (*n* = 8), unsteadiness (*n* = 8); four patients felt the need to make compensatory movements, one perceived an external perturbation (i.e., the sensation of being pushed or pulled). The duration of dizziness was mostly in the range of seconds (*n* = 9). As to the frequency of such dizziness episodes, most reported more than five times during the entire course of the condition (*n* = 11). Slow head movements elicited dizziness only in five patients. Some patients had attempted to reproduce the dizziness with repetitive head movements to determine the specific trigger and find out the possible origin on their own (Table 2).

### Socioeconomic impact of symptoms

Fourteen out of the 19 patients spontaneously complained about neck pain associated dizziness beyond the specific questions (see Table 2). Twelve patients had consulted more than one medical specialist, with an average of 2.6 specialist consultations. In four cases, the cervical spine was considered to be the source of the symptoms. Eight patients described a fear of falling; four, significant discomfort; 12 described the dizziness as subjectively impairing, and four would take sick leave due to dizziness. At the time of presentation, 10 had developed avoidance behavior such as no longer riding a bicycle/motorcycle, avoiding fast head movements or overhead work, no longer leaving their house unaccompanied, or doing any sports which involve head movement. Twelve described the dizziness as subjectively impairing, and 4 had been on sick leave due to dizziness.

### Head movement restrictions and neuro-orthoptic findings

In the clinical examination, the greatest limitation of cervical spine movement compared to the expected movement range indicated in previous literature [41] was observed for head extension, followed by horizontal rotation; mean flexion about the x-axis in the pitch plane of approx. 40.3° ± 10.9° (89% of expected); mean extension of 41.3° ± 20.5° (59% of expected); mean left rotation about the z-axis in the yaw plane of 56.5° ± 18.5° (71% of expected); mean right rotation of 56.8° ± 16.5° (71% of expected); mean left tilt about the y-axis in the frontal plane of 35.8° ± 9.3° (79% of expected); mean right tilt of 35.8° ± 10.3° (79% of expected).

Neuro-ophthalmological examinations did not reveal a peripheral or central disorder of the ocular motor or vestibular system. Fourteen patients exhibited mildly impaired smooth pursuit, which was considered age-appropriate in all patients; two displayed a mild gaze-evoked nystagmus; in one a head-shaking-nystagmus was inducible. No peripheral or central positional nystagmus, eye muscle or gaze paresis were observed in any. Six patients showed (near) exophoria, nine orthophoria, and two had congenital strabismus. Additional tests, such as the Video Head Impulse Test (vHIT) and caloric testing, were normal in all patients. Mild SVV deviations (2.9° and 5.3°) in binocular testing was observed in two patients. The only relevant pathological finding in standard posturography using an artificial neural network analysis was a pattern, typical for a functional (psychosomatic) stance disorder, in six patients.

## Stance and gait performance during active head-turn challenges

**Fig. 3:**
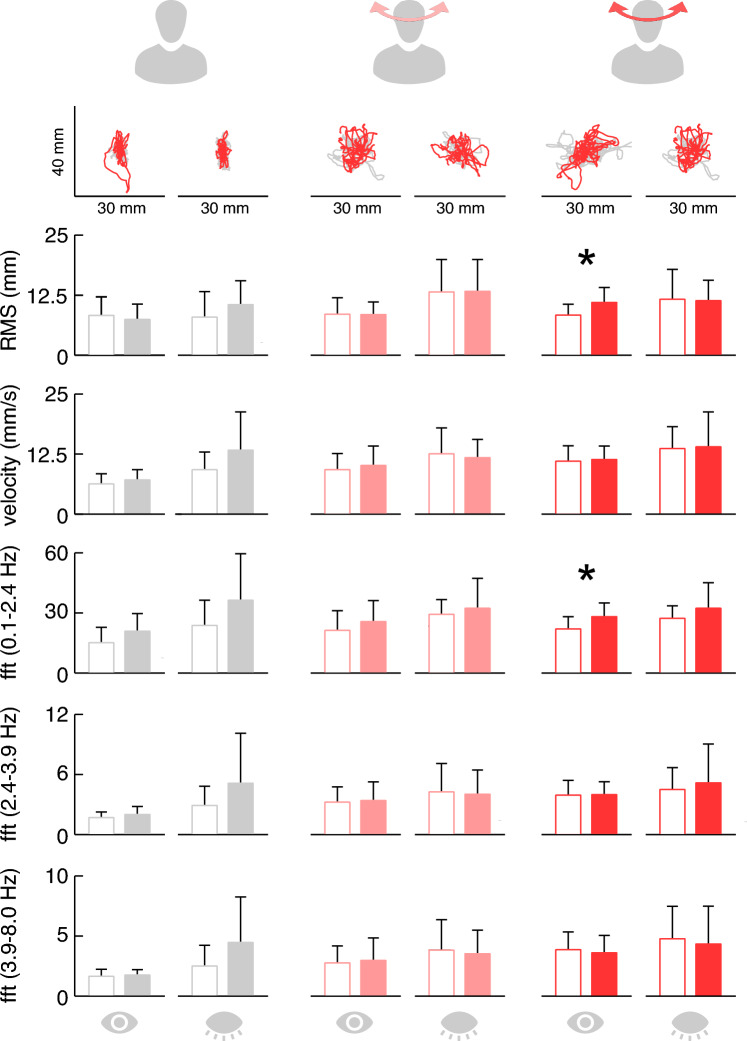
Stance performance of patients (filled bars) and healthy controls (white bars) during undisturbed quiet standing (left panel), standing with slow continuous active head turns (middle panel, light red) and fast active head turns (right panel, dark red) with eyes open or closed, respectively. Exemplary traces of one patient (red) and one control (grey) are presented on top of each condition (sway path of the center of pressure)

In a subgroup of 13 CD patients and 13 age-matched HS, the impact of painful neck stiffness on stance and gait performance under normal conditions without voluntary head movements and during active head-turn challenges was evaluated. Undisturbed quiet stance performance with eyes open was unremarkable in patients compared to controls, though there was greater variation of sway parameters in the patient group when standing with eyes closed. Slow continuous head turns similarly increased body sway in patients and controls (p < 0.001). During fast active head turns, however, static balance was more challenged in patients compared to controls in terms of an increase in the amplitude (RMS: *p* = 0.031) and low-frequency components of body sway (fft_low_: *p* = 0.040). These latter differences were only observed when patients executed the head turns with their eyes open (Fig. 3). Furthermore, the increase of sway amplitudes during fast active head movements was associated to the subjectively felt head motion restriction (*R* = 0.71; *p* = 0.010).

**Fig. 4:**
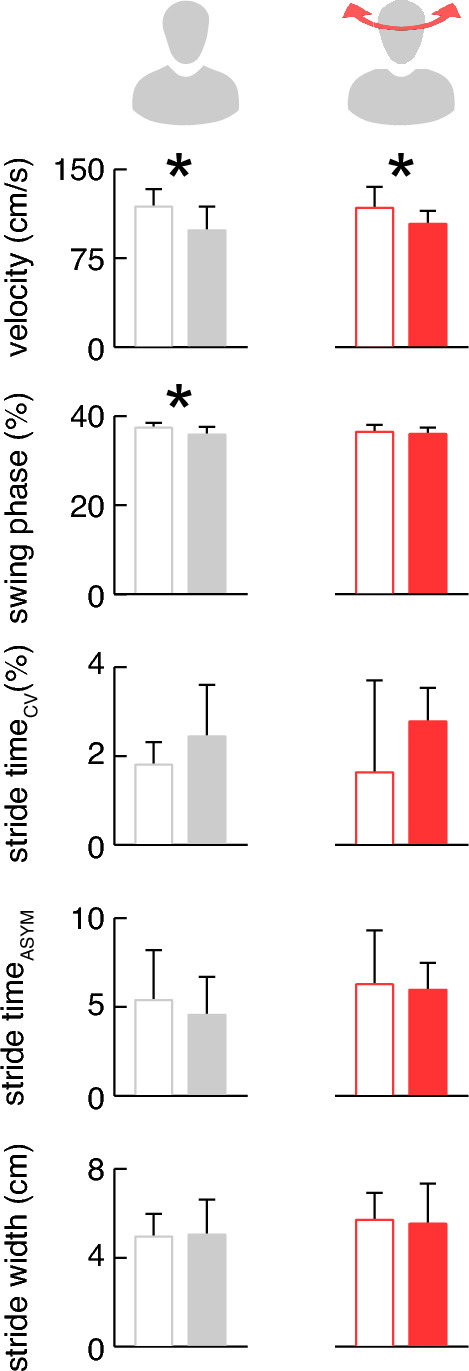
Walking performance of patients (filled bars) and healthy controls (white bars) during undisturbed gait (left panel) and while walking with fast active head turns (right panel, dark red)

Locomotion mobility in the group of patients were moderately impaired, in that they took longer to complete the TUG test (5.51 ± 0.96 s vs. 4.81 ± 0.62 s; *p* = 0.037), but their FGA assessment were within the norm (27.9 ± 3.1 points vs. 29.3 ± 1.3 points). This was also reflected in the patients’ undisturbed walking performance. However, individually preferred locomotion speed was characterized by a reduced velocity (*p* = 0.008) and shorter swing phases (*p* = 0.013) (Fig. 4). The reduced baseline gait speed was associated to the clinically observed pattern of a functional (psychosomatic) stance disorder (*R* = 0.63; *p* = 0.020).

The slowed gait pattern of patients persisted during challenged walking with active fast head turns (*p* = 0.027), which additionally impaired walking stability in patients and healthy controls equally in terms of increased walking variability, asymmetry and a broadened base of support (*p* = 0.004).

## Discussion

### Head motion-induced spells of cervical dizziness

Patients with vertigo or dizziness associated with chronic or frequently recurrent neck pain syndromes describe their symptoms spontaneously, quite generically, and imprecisely as dizziness, lightheadedness, drowsiness, or unsteadiness [21, 23, 46]. In the current study, we focused on head movement-induced dizzy episodes by asking more specific questions about typical triggers, duration of attacks, as well as perceptual and postural consequences. All of the patients—in addition to more general unspecific descriptions—had experienced short bursts of dizziness in the course of their condition, which was elicited irregularly by voluntary head movements, especially when it exacerbated their neck pain and caused restrictions of head movements. According to our earlier descriptions [13], the head motion-induced spells of dizziness were evoked by rapid—not by slow—head rotations particularly in the horizontal yaw plane, and less often by head flexion or extension in the pitch plane. The durations were usually short in the range of seconds with varying unpleasant perceptions, unsteadiness, fear of falling, and avoidance behavior.

### Effects of head movements on postural balance and locomotion

Chronic neck pain did not show any impact on free, upright stance with eyes open or closed. Slow horizontal head rotations increased body sway equally in both patients and healthy controls. In contrast, rapid head rotations resulted in a significantly more instable posture with increased sway ranges in patients compared to healthy controls. The latter effect was only observed with eyes open but not in the absence of visual feedback. Both the head-motion induced perception of short bursts of dizziness and their effect on postural balance are compatible with the hypothetical mechanism of a multisensory mismatch between the expected and the altered actual reafferences because of the painful movement restriction in the afflicted patients as previously discussed for patients with acute neck pain syndromes [13]. The explanation that only fast, but not slow head movements elicit significant postural instability in the patients (Fig. 3) depends on the different control of fast (saccadic) and slow head movements. In analogy to eye movements, a saccadic head movement is executed towards a certain target without control of velocity and amplitude during the movement, while slow movements can be continuously controlled in velocity and amplitude. This means that a mismatch between the expected and actual reafferences will only occur in fast head movements, unexpectedly limited by the neck pain.

Instrumented analysis of gait revealed that patients with chronic or recurrent forms of neck pain adopt a cautious pattern of walking [20] that is primarily characterized by slowed and careful mode of locomotion and commonly observed in anxious elderly individuals. These walking alterations were not specifically triggered by active head motions but already present during normal undisturbed walking. Hence, chronic or recurrent neck pain appears to more permanently affect walking than stance performance. The latter is also indicated by the finding of a reduced functional mobility in patients as assessed by the TUG test.

These patterns of automated stance and gait analysis in chronic of recurrent forms of neck pain with increased body sway and cautious locomotion closely resembled those of two other conditions: (1) individuals susceptible to visual height intolerance when exposed to heights [14, 26, 34] and (2) patients with phobic postural vertigo [11], further defined by the Bárány Society as persistent postural perceptional dizziness [39], which is also characterized by an increased body sway and reduced walking speed [32, 35, 47]. The diagnostic differentiation to visual height intolerance is not relevant, since the specific trigger is exposure to height. The differentiation of CD symptoms from phobic postural vertigo, however, is clinically relevant, since this psychosomatic condition is associated with fear of falling, avoidance behavior, and reduced walking speed. Cases of transitions from vestibular disorders to phobic postural vertigo have been described, showing an overlap [40]. This could explain the findings in standard posturography in some patients, which revealed a pattern typical for a functional stance disorder, possibly responsible for the large variation of the postural sway when the eyes were closed.

### Proprioceptive cervical dizziness

Cervical dizziness has been known by different names, such as cervicogenic vertigo or proprioceptive cervicogenic dizziness, because of numerous diagnostic and pathophysiological uncertainties [21]. The term “proprioception” was coined by Sherrington in 1906 [38] to describe the sensory information from joints and muscles necessary for the awareness of positions and movements of the body and body segments in space. This still inconsistently defined combination of sensors emerged from the “muscular sense” earlier proposed by Bell in 1826 [5] and by Bastian in 1887 [4] as “kinesthesia” [24]. The somatosensory focus of this term on the cervical spine and neck muscles is attractive but may neglect the importance of the visual and vestibular systems for the symptomatology of cervical dizziness. Input from muscle spindles converges with visual and vestibular motion signals for spatial orientation, gaze, and postural control [27]. The use of the term “proprioceptive cervical dizziness” should not refer to a particular sense, but to the functional achievement of a multisensory ensemble in which somatosensory, visual, and vestibular cues play an important role. When Sherrington coined the term in the early twentieth century, the function of the vestibular system had just been discovered by others. Sherrington in his work who was focused on spinal reflexes did not refer to the important contribution of visual and vestibular information for proprioception. However, the major contribution of sight/visual influence is compatible with our findings that the only significant impairment of postural sway during rapid head movements was seen in the patients, who performed the task with their eyes open.

### Limitations

The finding that six of the patients with chronic neck pain syndrome exhibited the typical pattern of functional vertigo newly defined by the Bárány Society as persistent postural perceptual dizziness [39] is important since the transition of acute dizziness syndromes to functional disorders has been shown [15]. Furthermore, this transition and overlap between cervical and functional dizziness is clinically relevant and explains the diversity of the complaints of the patients and the literature.

The differential effects of slow and fast head movements on postural balance could only be tested by posturography, while analysis of locomotion on the gait carpet was only possible with slow head movements due to the risk of falling.

## Supplementary Information

Below is the link to the electronic supplementary material.

Supplementary file1 (DOCX 25 kb)

## Data Availability

The datasets used and/or analyzed during the current study will be available from the corresponding author upon reasonable request.
