# Supplementary material for: Impact of proprioceptive cervical dizziness in chronic neck pain syndromes on gait and stance during active head-turn challenges
Source: J Neurol. 2024 Oct 15;271(12):7460–70. doi: 10.1007/s00415-024-12711-8 (PMC11588840; doi:10.1007/s00415-024-12711-8)
Supplement: Supplementary file 1 — Supplementary file1 (DOCX 25 kb) [file 415_2024_12711_MOESM1_ESM.docx]

**Supplementary Table: Medical history and clinical examination form**

This table includes the Neck Pain and Disability Scale (selected and modified questions) and additional questions on medical history taking and results of physical examination

| Cervical syndrome | ⃝ Right | | | | ⃝ Left | | | | ⃝ Both sides | | | |
| --- | --- | --- | --- | --- | --- | --- | --- | --- | --- | --- | --- | --- |
| Onset | ⃝ Acute (days) | | | ⃝ Subacute (weeks) | | | ⃝ Chronic (months/years) | | | ⃝ Subacute/recurrent | | |
| Cause | Please indicate:­­­­­­­­__________________________________________________ | | | | | | | | | | | |
| Prior findings on examination regarding cervical syndrome | | | | | | | | | | | | |
| Orthopedic | Please indicate:­­­­­­­­__________________________________________________ | | | | | | | | | | | |
| Neurological | Please indicate:­­­­­­­­__________________________________________________ | | | | | | | | | | | |
| Spinal column (X-ray / MR) | Please indicate:­­­­­­­­__________________________________________________ | | | | | | | | | | | |
| Movement restriction | | | | | | | | | | | | |
| Localisation of main dysfunction segment | Please indicate:­­­­­­­­__________________________________________________ | | | | | | | | | | | |
| Movement ranges (in degrees) |  | | | | | | | | | | | |
| F (flexion) | Please indicate:­­­­­­­­__________________________________________________ | | | | | | | | | | | |
| E (extension) | Please indicate:­­­­­­­­__________________________________________________ | | | | | | | | | | | |
| Rl (left rotation) | Please indicate:­­­­­­­­__________________________________________________ | | | | | | | | | | | |
| Rr (right rotation) | Please indicate:­­­­­­­­__________________________________________________ | | | | | | | | | | | |
| Sl (left lateral tilt) | Please indicate:­­­­­­­­__________________________________________________ | | | | | | | | | | | |
| Sr (right lateral tilt) | Please indicate:­­­­­­­­__________________________________________________ | | | | | | | | | | | |
| Neck Pain and Disability Scale | | | | | | | | | | | | |
| How severe is your neck pain today? | 0 (none/no)  ⃝ | 1  ⃝ | | | 2  ⃝ | | 3  ⃝ | | 4  ⃝ | | | 5 (max/ always)  ⃝ |
| How bad is your neck pain when it is at its worst? | 0 (none/no)  ⃝ | 1  ⃝ | | | 2  ⃝ | | 3  ⃝ | | 4  ⃝ | | | 5 (max/ always)  ⃝ |
| How bad is the neck pain with walking? | 0 (none/no)  ⃝ | 1  ⃝ | | | 2  ⃝ | | 3  ⃝ | | 4  ⃝ | | | 5 (max/ always)  ⃝ |
| Does the neck pain affect your day-to-day activities? | 0 (none/no)  ⃝ | 1  ⃝ | | | 2  ⃝ | | 3  ⃝ | | 4  ⃝ | | | 5 (max/ always)  ⃝ |
| Does your neck pain interfere with personal care (eating or personal hygiene as bathing, dressing, etc.)? | 0 (none/no)  ⃝ | 1  ⃝ | | | 2  ⃝ | | 3  ⃝ | | 4  ⃝ | | | 5 (max/ always)  ⃝ |
| How much has your neck pain changed your outlook on life and your future? | 0 (none/no)  ⃝ | 1  ⃝ | | | 2  ⃝ | | 3  ⃝ | | 4  ⃝ | | | 5 (max/ always)  ⃝ |
| Does your neck pain affect your feelings? | 0 (none/no)  ⃝ | 1  ⃝ | | | 2  ⃝ | | 3  ⃝ | | 4  ⃝ | | | 5 (max/ always)  ⃝ |
| How stiff is your neck? | 0 (none/no)  ⃝ | 1  ⃝ | | | 2  ⃝ | | 3  ⃝ | | 4  ⃝ | | | 5 (max/ always)  ⃝ |
| How greatly is the mobility of your head impaired? | 0 (none/no)  ⃝ | 1  ⃝ | | | 2  ⃝ | | 3  ⃝ | | 4  ⃝ | | | 5 (max/ always)  ⃝ |
| How well do painkillers help with your neck pain? | 0 (none/no)  ⃝ | 1  ⃝ | | | 2  ⃝ | | 3  ⃝ | | 4  ⃝ | | | 5 (max/ always)  ⃝ |
| On a scale from 0 (no pain) to 10 (maximal pain imaginable), please rate your spontaneous neck pain. | 0 1 2 3 4 5 6 7 8 9 10  ⃝ ⃝ ⃝ ⃝ ⃝ ⃝ ⃝ ⃝ ⃝ ⃝ ⃝ | | | | | | | | | | | |
| On a scale from 0 (no pain) to 10 (maximal pain imaginable), please rate your neck pain during head movement. | 0 1 2 3 4 5 6 7 8 9 10  ⃝ ⃝ ⃝ ⃝ ⃝ ⃝ ⃝ ⃝ ⃝ ⃝ ⃝ | | | | | | | | | | | |
| Medical history | | | | | | | | | | | | |
| Vertigo disorders? (Menière's disease, Benign paroxysmal positional vertigo, Bilateral vestibulopathy, Unilateral vestibulopathy/vestibular neuritis, Phobic postural vertigo/functional vertigo, Vestibular migraine, Vestibular paroxysmia, other) | ⃝ Yes | | | | | | ⃝ No | | | | | |
| Migraine attacks? | ⃝ Yes | | | | | | ⃝ No | | | | | |
| Neurological diseases? (Cerebellar ataxia, Parkinson's diseases, Polyneuropathy, other) | ⃝ Yes | | | | | | ⃝ No | | | | | |
| Sedative drugs/other psychotropic drugs? | ⃝ Yes | | | | | | ⃝ No | | | | | |
| Specific questions about cervical vertigo/dizziness (consider this carefully, as you may have experienced it but not attached any particular significance to it) | | | | | | | | | | | | |
| 1. During the time with the acute neck pain, did you experience a surprising short bout of dizziness/steadiness once or more during rapid head movements? | ⃝ Yes | | | | ⃝ Don‘t know | | | | ⃝ No | | | |
| Only answer the following questions if you answered "yes" or "don't know" to the previous question. | | | | | | | | | | | | |
| 2. Did the dizziness occur… | | | | | | | | | | | | |
| …when turning your head? | ⃝ Right | | | ⃝ Left | | | ⃝ Both Sides | | | ⃝ I dont know | | |
| …when bending your head? | ⃝ Forward | | | | ⃝ Backward | | | | ⃝ I dont know | | | |
| …when sitting? | ⃝ Yes | | | | | | ⃝ No | | | ⃝ I dont know | | |
| …when lying or lying down? | ⃝ Yes | | | | | | ⃝ No | | | ⃝ I dont know | | |
| 3. What did you feel? *(multiple answers possible)* | ⃝ Drowsiness | | ⃝ Spinning vertigo | | | ⃝ Unsteady on your feet | | ⃝ Compensatory movement (head, body, stance) | | | ⃝ Feeling as if you are being pushed (app. perturbation) | |
| 4. How long did the dizziness last? | ⃝ Seconds (fractions/1/2/more) | | | | ⃝ Minutes | | | | ⃝ Hours | | | |
| 5. How often did it occur? | ⃝ 1 time | | | ⃝ 2 to 5 times | | | ⃝ More often | | | ⃝ Daily | | |
| 6. Did you also have dizziness during slow head movements? | ⃝ Yes | | | | | | ⃝ No | | | | | |
| 7. Did you try other head movements after an attack to find out if it occurs regularly? | ⃝ Yes | | | | | | ⃝ No | | | | | |
| 8. How did you explain the dizziness? | ⃝ Cervical spine | | | ⃝ Circulation | | | ⃝ I was wrong | | | ⃝ Other: _______ | | |
| 9. Were there other body movements that caused the same dizziness? | ⃝ Yes | | | | | | ⃝ No | | | | | |
| 10. Did the dizziness occur only during body activity (when standing/walking) or also at rest (when sitting/lying)? |  | | | | | | | | | | | |
| Standing/walking | ⃝ Yes | | | | | | ⃝ No | | | | | |
| Sitting/lying | ⃝ Yes | | | | | | ⃝ No | | | | | |
| Other situations? | ⃝ Yes | | | | | | ⃝ No | | | | | |
| If yes, which? |  | | | | | | | | | | | |
| 11. Did the dizziness occur only during the time with the neck pain or also after the pain went away? | ⃝ Only during neck pain | | | | ⃝ Also after the pain went away | | | | ⃝ How long afterwards:__________ | | | |
| 12. Did the dizziness occur mainly at the beginning of the neck pain? *(multiple answers possible)* | ⃝ In the first few days | | | | ⃝ After weeks | | | | ⃝ Also after months | | | |
| 13. Had you experienced this type of vertigo before? | ⃝ Yes | | | | | | ⃝ No | | | | | |
| 14. Have you been free of vertigo since the neck pain went away? | ⃝ Yes | | | | | | ⃝ No | | | | | |
| If no: | | | | | | | | | | | | |
| How long have you had no neck pain? | Please indicate:­­­­­­­­__________________________________________________ | | | | | | | | | | | |
| Did the dizziness improve overall after the neck pain subsided? | ⃝ Yes | | | | | | ⃝ No | | | | | |
| if yes: to what extent? |  | | | | | | | | | | | |
| 15. Did you mention the dizziness as a complaint without being asked specifically about it? | ⃝ Yes | | | | | | ⃝ No | | | | | |
| If yes, to whom? | | | | | | | | | | | | |
| ENT doctor | ⃝ | | | | | | | | | | | |
| Neurologist | ⃝ | | | | | | | | | | | |
| Orthopedist | ⃝ | | | | | | | | | | | |
| Specialist in internal medicine | ⃝ | | | | | | | | | | | |
| General practitioner | ⃝ | | | | | | | | | | | |
| Ophthalmologist | ⃝ | | | | | | | | | | | |
| Other doctor/therapist | ⃝ | | | | | | | | | | | |
| What was the diagnosis? | Please indicate:­­­­­­­­__________________________________________________ | | | | | | | | | | | |
| 16. How disturbing/impairing is the vertigo for you? |  | | | | | | | | | | | |
| Fear of falling? | ⃝ Yes | | | | | | ⃝ No | | | | | |
| Other fears of illness? |  | | | | | | | | | | | |
| 17. What impact does vertigo have on your daily life? |  | | | | | | | | | | | |
| Is there anything you avoid doing? | ⃝ Yes | | | | | | ⃝ No | | | | | |
| If yes, please describe. | Please indicate:­­­­­­­­__________________________________________________ | | | | | | | | | | | |
| Other effects? | Please indicate:­­­­­­­­__________________________________________________ | | | | | | | | | | | |
| Does the vertigo affect you subjectively? | ⃝ Yes | | | | | | ⃝ No | | | | | |
| If yes, to what extent? | ⃝ minimal | | | ⃝ mild | | | ⃝ moderate | | | ⃝ severe | | |
| 18. Have you already been on sick leave because of the vertigo? | ⃝ Yes | | | | | | ⃝ No | | | | | |
| Orthoptic examination findings | | | | | | | | | | | | |
| Smooth pursuit | Please indicate:­­­­­­­­__________________________________________________ | | | | | | | | | | | |
| End-position nystagmus/direction of gaze nystagmus | Please indicate:­­­­­­­­__________________________________________________ | | | | | | | | | | | |
| Positional nystagmus | Please indicate:­­­­­­­­__________________________________________________ | | | | | | | | | | | |
| Gaze paresis/eye muscle paresis | Please indicate:­­­­­­­­__________________________________________________ | | | | | | | | | | | |
| Eye position (phoria/tropia/other) | Please indicate:­­­­­­­­__________________________________________________ | | | | | | | | | | | |
| Additional examinations | | | | | | | | | | | | |
| Video head impulse test | Please indicate:­­­­­­­­__________________________________________________ | | | | | | | | | | | |
| Posturography | Please indicate:­­­­­­­­__________________________________________________ | | | | | | | | | | | |
| Gait analysis | Please indicate:­­­­­­­­__________________________________________________ | | | | | | | | | | | |
